# Socio-economic status as predictors of malaria transmission in KwaZulu-Natal, South Africa. A retrospective study

**DOI:** 10.4314/ahs.v22i2.24

**Published:** 2022-06

**Authors:** Osadolor Ebhuoma, Michael Gebreslasie, Ropo Ebenezer Ogunsakin

**Affiliations:** 1 Geography and Environmental Sciences, School of Agricultural, Earth, and Environmental Sciences, University of KwaZulu-Natal, Westville Campus, Durban, 4000, South Africa; 2 Biostatistics, Discipline of Public Health Medicine, School of Nursing and Public Health, University of KwaZulu-Natal, Howard College Campus, Durban, 4041, South Africa

**Keywords:** malaria, socialeconomic status, Bayesian modelling, KwaZulu-Natal, South Africa

## Abstract

**Background:**

Understanding the socioeconomic status that influences malaria transmission in KwaZulu-Natal, South Africa is vital in creating policies and strategies to combat malaria transmission, improve socioeconomic conditions and strengthen the malaria elimination campaign.

**Objectives:**

To determine the relationship between socioeconomic status and malaria incidence in KwaZulu-Natal, South Africa.

**Methods:**

Socioeconomic information (gender, age, no formal education, no electricity, no toilet facilities, unemployment) and malaria data for 2011 were obtained from Statistics South Africa and the malaria control program of KwaZulu-Natal, South Africa respectively. The analysis was conducted employing the Bayesian multiple regression model.

**Results:**

The obtained posterior samples show that all the variables employed in this study were significant and positive predictors of malaria disease at 95% credible interval. The low socioeconomic status that exhibited the strongest association with malaria risk was lack of toilet facilities (odd ratio =12.39; 95% credible interval = 0.61, 24.36). This was followed by no formal education (odd ratio =11.11; 95% credible interval = 0.51, 24.10) and lack of electricity supply (odd ratio =8.94; 95% credible interval = 0.31, 23.21) respectively.

**Conclusions:**

Low socioeconomic status potentially sustains malaria transmission and burden. As an implication, poverty alleviation and malaria intervention resources should be incorporated side by side into the socioeconomic framework to attain zero malaria transmission.

## Introduction

**Figure 1:**
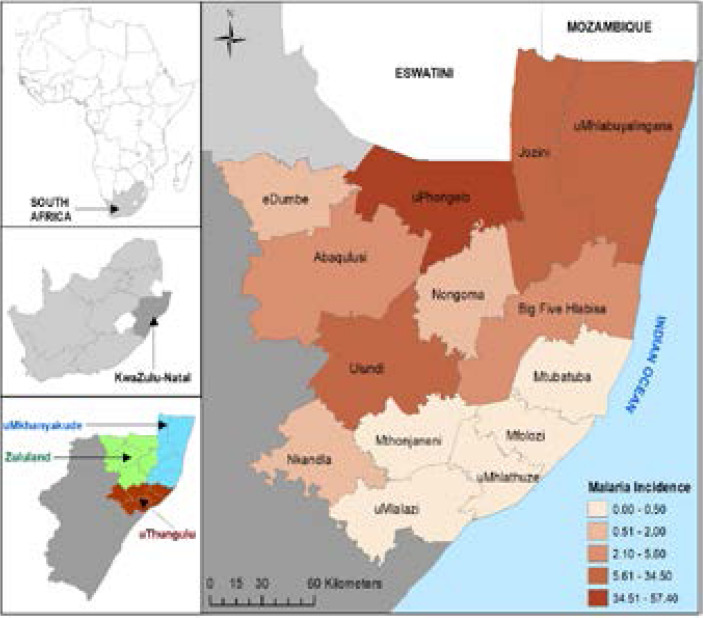
Study area and the spatial distribution of malaria incidence in uMkhanyakude, Zululand and uThungulu

Malaria is endemic in the north-eastern part of KwaZulu-Natal (KZN) province, South Africa (SA) 1, 2. Low malaria incidence and zero malaria incidence have been recorded in the malarious local municipalities in KZN (see Figure 1) arising from the influence of efficient malaria control and intervention strategies^1^, ^3^. The malaria control and elimination strategy in SA focuses on targeted and timely indoor residual spraying, enhanced surveillance systems (this includes integrated malaria system, quality data management system, passive and active case detection, routine entomological surveillance and larviciding), treatment with antimalarials, community health campaigns and door-to-door health education, ongoing operational research, evidence-based policies and enhanced inter-district and cross-border collaborations with neighbouring countries (including Botswana, Kingdom of Eswatini, Mozambique, and Namibia 3.

Several studies have focused on the implications of the interventions mentioned above on malaria disease transmission and also have suggested ways that they can be utilised optimally 4–8. Less attention has been paid to understand the influence socioeconomic status (SES)/factors have on malaria transmission. Although, the KZN malaria control and intervention strategies have led to commendable malaria transmission control and zero transmission mainly in urban areas 3, 9, malaria transmission is still prevalent in some semi-urban and rural areas 3. At a time when the KZN health department is putting more effort to eliminate malaria, it is crucial to investigate and identify relevant factors of malaria transmission in the province 3.

An understanding of the SES of a population is important because it can result in identifying favourable settings for mosquito proliferation and malaria transmission, and population groups more susceptible to malaria disease, including but not limited to children, elderly and gender-related risks due to settings and lifestyles. These assertions have been communicated extensively in various studies conducted in different malaria-endemic settings^10^–^17^. Most of the studies employed models based on the classical theory to identify relevant socioeconomic predictors of malaria transmission. However, the Bayesian modelling approach has been applied by different authors in diverse settings 15, 18–21, and suggested that it is a good alternative to the classical approach 22–24.

The Bayesian modelling approach combines prior distribution or information with the traditional likelihood to derive the posterior distribution of the parameter(s) of interest on which the statistical inference is based using the Markov chain Monte Carlo (MCMC) methods such as the Metropolis-Hastings algorithm and Gibbs sampler^23^. In contrast, the classical approach depends only on the likelihood to derive the statistical inference. Accordingly, this study aims to provide knowledge on the SES risk factors for malaria transmission in KZN, SA, employing the Bayesian method. It can equip the relevant authorities and policymakers with the necessary information to improve socioeconomic conditions and implement appropriate malaria intervention strategies in addition to the pre-existing ones.

## Methods

### Study area

The study was based on data from the malarious local municipalities of uMkhanyakude, uThungulu and Zululand district municipalities in KZN province, SA, and these local municipalities are located in the north-eastern part of KZN (Figure 1). The study area limits to the north with the Kingdom of Eswatini and the republic of Mozambique, to the east stretching down to the southeast with the Indian Ocean, and to the west with other local municipalities in KZN.

### Data

In this study, we obtained and used data from two separate sources. The database of daily records of clinically confirmed malaria cases for 2011 was obtained from the malaria control program of KZN, SA and aggregated at the local municipality level for uMkhanyakude, uThungulu and Zululand district municipalities. Figure 1 shows the spatial distribution of malaria incidence in the respective local municipalities in 2011. We obtained the database of socioeconomic and demographic information for the study area from Statistics SA (Census 2011 Municipal report - KZN, SA) 25. This database is freely available to the general public, and it is protected by scientific and ethical clearance and authorisation. The demographic and socioeconomic variables used for this study were: gender, age group (less than 5years old, 5 to 14 years old, 15 to 34 years old, 35 to 65 years old, over 65 years old) no formal education, no electricity, no toilet facilities, unemployment. We used the 2011 dataset (SES and malaria incidence) for this study because it completely covered the study area, and the last census in SA was conducted in 2011.

### Model/Analysis

To determine the association between the explanatory variables (demographic and socioeconomic variables) and the dependent variable (malaria incidence by year) at the local municipality level, a Bayesian multiple regression model was formulated to obtain the posterior samples via a Markov chain Monte Carlo (MCMC) methodology. The analysis was conducted using the WinBUGS software.

### Bayesian multiple regression model formulation

A regression model comprises (1) dependent variable(s), which represents the stochastic part whose effect is uncertain before the analysis. (2) The explanatory variable(s), which represents the non-stochastic or fixed parts and (3) a parameter that links the two set of variables. The model can be expressed as 23

(1) $${\text{Y|X}}_{\text{01}},{\text{X}}_{\text{02}},.........{\text{X}}_{\text{p}}\text{α(}\theta \text{)}$$

where Y is 1t he dependent variable, X_01_, X_02_,........., X_p_ are the explanatory variables which are specified as whole set of real numbers, and α(***θ***) is a distribution with parameter vector ***θ***.

The simplest and commonly used distribution for the regression model is a normal distribution, and it can be written as

(2) $${\text{Y|X}}_{\text{01}},{\text{X}}_{\text{02}},.........{\text{X}}_{\text{p}}\mathrm{normal}{\text{(μ,σ}}^{\text{2}}\text{)}$$

where *Normal*(μ,σ^2^) is the distribution, μ is mean, and σ^2^ is variance.

While a multiple regression model with a single dependent variable (univariate), having a normal distribution with mean and variance can be summarised and rewritten as 23

(3) $${\text{Y|X}}_{\text{01}},{\text{X}}_{\text{02}},.........{\text{X}}_{\text{p}}\mathrm{normal}{\text{ (μ,(β,X}}_{\text{01}}{\text{,X}}_{\text{02}}\text{,}.........{\text{,X}}_{\text{p}}{\text{)σ}}^{\text{2}}\text{)}$$

with

(4) $${\text{μ (β, X}}_{\text{01}}{\text{,X}}_{\text{02}}\text{,}.........{\text{,X}}_{\text{p}}{\text{) = β}}_{\text{01}}+{\text{β}}_{\text{02}}{\text{X}}_{\text{01}}+........+{\text{β}}_{\text{p}}{\text{X}}_{\text{p}}={\text{β}}_{\text{01}}+\sum_{\text{i=01}}^{\text{p}}{\text{β}}_{\text{j}}{\text{X}}_{\text{i}}$$

where and σ^2^ and β = (β_01_,β_02_,........., β_p_)^T^ are the set of regression parameters under estimation.

To specify the model in WinBUGS, the likelihood function for the observed sample and the prior information (or prior distribution) for the parameters are required. Thus, the likelihood function (extracted from equations (3) and (4)) is expressed as 23, 26

(5) $${\text{Y}}_{\text{i}}\mathrm{normal}{\text{ (μ}}_{\text{i}}{\text{,σ}}^{\text{2}}\text{)}$$

(6) $${\text{μ}}_{\text{i}}={\text{β}}_{\text{01}}+{\text{β}}_{\text{02}}{\text{x}}_{\text{i1}}+........+{\text{β}}_{\text{p}}{\text{x}}_{\text{ip}}\text{ for i = 01, }........\text{, n}$$

While the prior distribution for all the parameters are assumed to have the structure

(7) $$\text{S(β, τ) = }\prod_{\text{j=01}}^{\text{p}}{\text{S(β}}_{\text{j}}\text{)}\text{ S(τ),}$$

β_j_ ∼ *normal* (μβ_i_, *R*_j_^2^) for j = 01, 02, 03..........,p

and


(8) τ~gamma (a,b)

From the above, the normal distribution is selected to 1 exhibit the prior information for *β* and the gamma distribution for the precision parameter, τ = σ^-2^.

However, we set up a uniform prior distribution for our regression model due to its subjective nature 23. Therefore, the formulated uniform prior distribution we employed in our analysis is given as

β_j_ ∼ *uniform* (0.0, 25) for j = 01, 02, 03..........,p

and


(9) τ~gamma (0.001,0.001)

### MCMC implementation and convergence

The likelihood and the priors were used to obtain the posterior distributions and results of the model parameters via the MCMC approach known as the Gibbs sampling technique. The MCMC approach we adopted is described in details elsewhere 23. For our model, we assumed flat but proper priors as expressed in equation (8). We ran three parallel chains (with different starting points) for 50,000 iterations of the MCMC with a burn-in period of 1,000 iterations and a thinning interval of 5. Monitoring the convergence of the algorithm (i.e. assessing if the algorithm attained its equilibrium or target distribution) is vital for obtaining the posterior samples of all the monitored parameters (regression coefficients). It also indicates if the posterior samples came from the right target distribution. Thus, to ascertain if convergence was reached in our model, we employed four different diagnostic tests. These are the Monte Carlo (MC) errors calculation of all the regression and precision parameters, assessing the autocorrelation plot, the trace plot and the Gelman-Rubin covergence diagnostic test. The Gelman-Rubin convergence diagnostic test was employed because more than one chain (three parallel chains) were generated simultaneously.

## Results

### Spatial distribution of malaria incidence

The spatial distribution of malaria data of all the local municipalities obtained from the malaria control program of KZN, SA showed that malaria incidence ranged from 0 to 57.40 during 2011 (Figure 1). About 72% of the total malaria incidence was reported from uPhongolo, Jozini and uMhlabuyalingana local municipalities reported in 2011 were observed to be clustered in the areas neighbouring Mozambique and Kingdom of Eswatini. uPhongolo local municipality, which recorded the highest incidence of malaria (about 37%) shared borders with Kingdom of Eswatini and Mpumalanga province northwards (Figure 1).

### Posterior distribution of predictors associated with increased risk of malaria disease

In interpreting the posterior statistics, it is vital to note that a regression parameter with a positive posterior mean, exhibited a significant positive relationship with malaria incidence at 95% credible interval (Cred. I) if the interval does not include zero (0) (Table 1). While a regression parameter with negative posterior mean and interval containing zero (0), suggested an inverse relationship. But from our results in Table 1 regression, parameters with negative posterior mean were not observed.

**Table 1:** Posterior summary statistics for the multiple regression model

Regression parameter	SD	2.5%	Estimate	97.5%	MC error
Male	5.803	0.195	6.878	21.54	0.03163
Female	5.504	0.1754	6.393	20.58	0.03165
Less than 5 years old	7.066	0.4387	10.79	24.06	0.04225
5 to 14 years old	6.797	0.3274	9.413	23.58	0.04158
15 to 34 years old	6.273	0.2384	7.905	22.6	0.03568
35 to 65 years old	6.807	0.3322	9.556	23.63	0.04156
Over 65 years old	7.185	0.5758	11.93	24.28	0.04389
No formal education	7.132	0.5098	11.11	24.1	0.04195
No electricity	6.598	0.3073	8.941	23.21	0.03828
No toilet facilities	7.229	0.613	12.39	24.36	0.04041
Unemployment	6.253	0.2472	7.942	22.56	0.03671

**Precision** **parameter**					

tau	0.001026	5.36E-04	0.001981	0.004456	6.43E-06

SD= Standard deviation; MC error= Monte Carlo error

The posterior summary statistics (Table 1) obtained from the Bayesian multiple regression model analysis suggests that all the variables employed in the model are significant and positive predictors of malaria at 95% Cred. I. However, in terms of gender, males (odds ratio (OR) =6.88; 95% Cred. I =0.2, 21.54) were slightly at a higher risk of contracting malaria disease as compared to females (OR =6.39; 95% Cred. I = 0.18, 20.58) in the study population. Considering the different age groups, children less than five years old (OR =10.79; 95% Cred. I = 0.44, 24.06) and adults over 65years old (OR =11.93; 95% Cred. I = 0.58, 24.28) are the most vulnerable to malaria parasitaemia.

From the variables or predictors that represents low SES used in this study population, lack of toilet facilities (OR =12.39; 95% Cred. I = 0.61, 24.36) exhibited the strongest association with malaria and highest risk of malaria disease. This was followed by no formal education (OR =11.11; 95% Cred. I = 0.51, 24.10) and lack of electricity supply (OR =8.94; 95% Cred. I = 0.31, 23.21) respectively. While unemployment was identified as the weakest significant variable as compared to the other variables that represent low SES.

### MCMC output and convergence diagnostics

**Figure 2:**
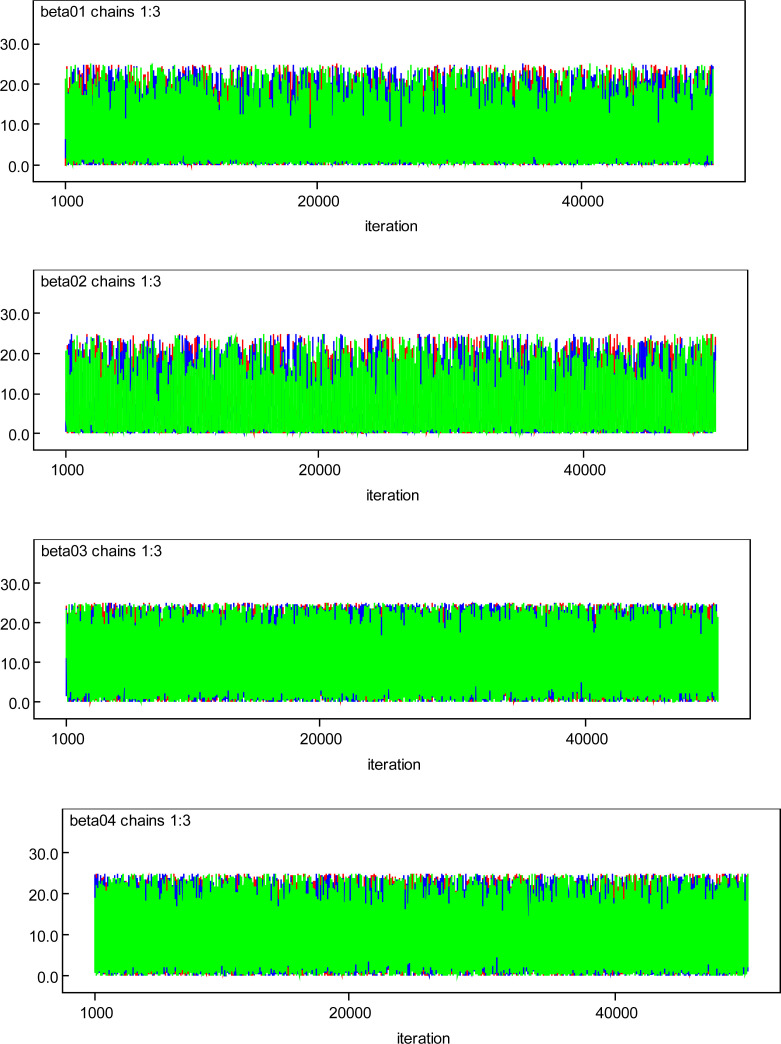
Trace plots for some selected independent parameters

The MCMC output results indicated that in obtaining the posterior samples of all the monitored parameters (regression coefficients and precision parameter), no convergence issue was experienced. The visual inspection of the trace plots or simulation plot for selected regression parameters (beta01=Male; beta02=Female; beta03=less than 5 years old; beta04=5 to 34 years old) presented in Figure 2 reveals that all the generated values were within a parallel zone, and notable patterns were not observed. Thus, the simulation is uniform throughout the plot.

**Figure 3:**
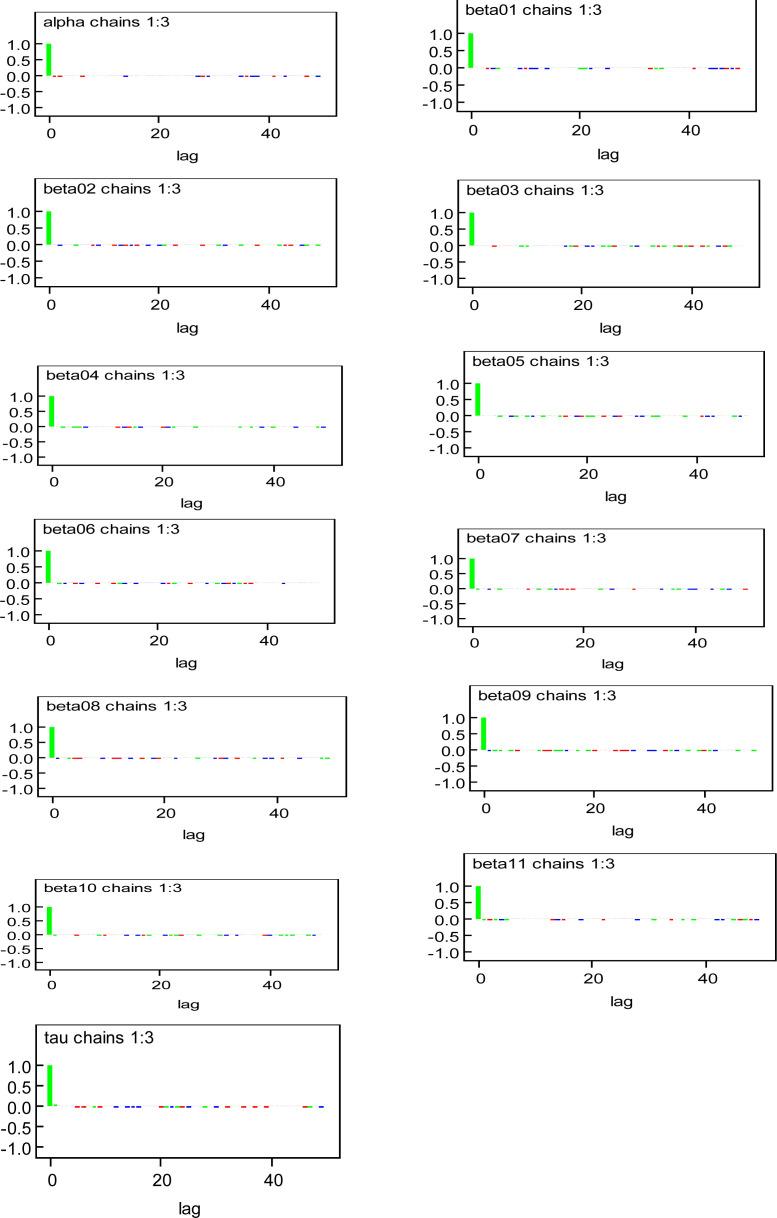
Autocorrelation function plot for the regression and precision parameters

The autocorrelations functions for all the monitored parameters (beta01=Male; beta02=Female; beta03=less than 5years old; beta04=5 to 14 years old; beta05=15 to 34 years old; beta06=35 to 65 years old; beta07=over 65 years old; beta08=no formal education; beta09=no electricity; beta10=no toilet facilities; beta11=unemployment) decayed sharply and were low (Figure 3). This indicates quick mixing of the chains and fast convergence to the posterior distributions 23, 27. At this point, we assumed convergence was reached. But we continued with the diagnostics and observed that the value of the MC errors calculated for the regression and precision parameters are very small compared to their corresponding estimated posterior standard deviations (SD). In other words, the MC error is less than 5% of the corresponding estimated posterior SD (See Table 1). This indicates the posterior mean was estimated with high precision and convergence was also assumed 23. Finally, we implemented a formal convergence diagnostic by using the Gelman and Rubin plots and the shrink factor (also known as the scale reduction point estimate) by using three different chains with three different initial points.

**Figure 4:**
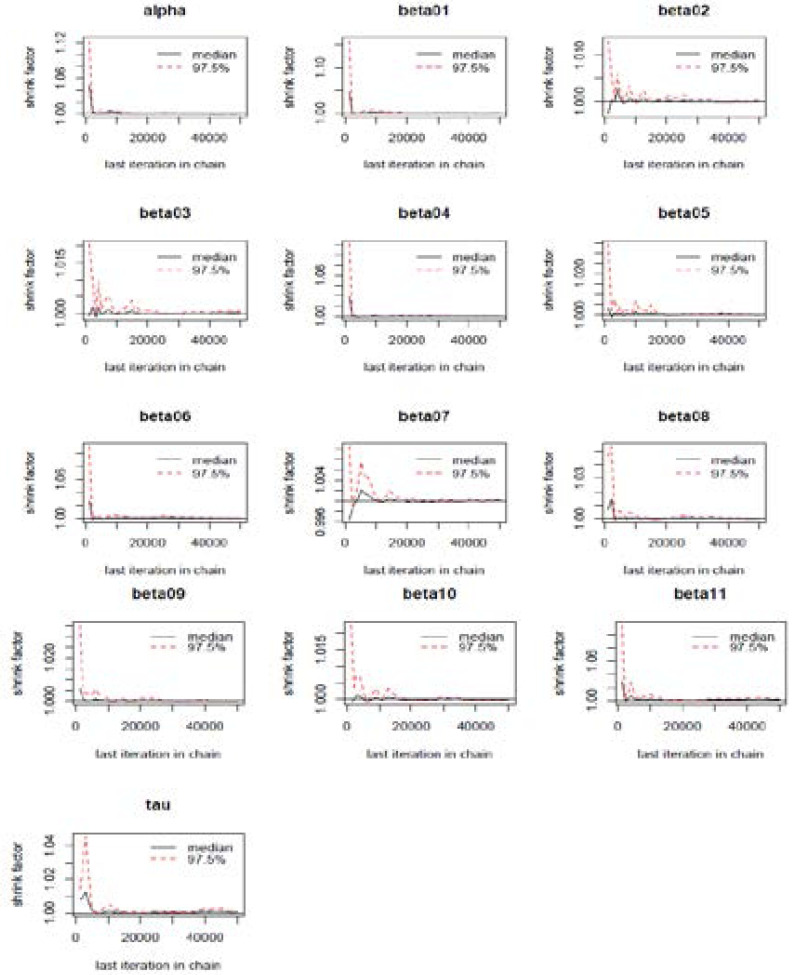
Gelman and Rubin shrink factor plots for the regression and precision parameters

From the Gelman and Rubin shrink factor plots illustrated in Figure 4, the shrink factor for all the monitored parameter (beta01=Male; beta02=Female; beta03=less than 5years old; beta04=5 to 14 years old; beta05=15 to 34 years old; beta06=35 to 65 years old; beta07=over 65 years old; beta08=no formal education; beta09=no electricity; beta10=no toilet facilities; beta11=unemployment) got to 1 abruptly as the number of iterations increases. At this point, we can confidently say convergence was reached, and we are also sampling from right posterior means 28.

## Discussion

In the local municipalities endemic to malaria in KZN, different low SES contribute to malaria risk transmission. In the order of highest malaria risk transmission, they include lack of toilet facilities, lack of formal education, lack of electricity, and unemployment. The contributory mechanism between these low SES and malaria disease runs side by side, which, in turn, can result in a vicious cycle of poverty. Our findings correspond with some previous studies conducted in India, South America, Cameroon, Ethiopia and Nigeria in which households without toilet facilities, unemployment, illiteracy and lack of electricity were are higher risks of contracting malaria compared to those exposed to medium and high SES 16, 29–33. However, these findings are contrary to the study conducted by Somi et al. 34 in Tanzania who found no relationship between malaria and SES. Also, the study conducted by Obaldia^30^, showed that lack of electricity and unemployed were not significantly (p < 0.05) association with malaria transmission.

Taking into account the epidemiological nature of malaria, and its relationship with the environment, it should not be surprising that lack of toilet facilities has the highest risk of being exposed to malaria as compared to other predictors that represent low SES of malaria assessed in this study. This demonstrates the heightened risk and exposure to malaria disease in households without decent and safe toilet facilities, and as such, open defecation is practised. Consequently, increased malaria transmission risk is unavoidable due to numerous and daily exposure to exophagic mosquito vectors and potential breeding sites from outdoor defecation. This is consistent with the findings by Ayele et al. 16 and Ayele et al. 33, who suggests that households without toilet facilities in Amhara, Oromiya and Southern Nation Nationalities and People regions of Ethiopia were more likely to test positive for malaria disease. On the contrary, no association was reported in Panama 30 and in the Brazilian Amazon 35.

Another significant predictor of malaria identified in this study is the lack of formal education. This can be attributed to weak knowledge and understanding of malaria transmission and prevention, and mosquito's resting behaviour and breeding. This suggests that the lack of formal education influences the success of the malaria elimination programme in KZN. Thus, strengthening of community health education and setting up communication activities to further equip the uneducated and also those with a low level of education in KZN about malaria may result in better application of intervention resources, and reduce the risk of malaria transmission. This can be achieved by conducting collaborative surveys with the relevant communities about knowledge, attitudes and practices that drive malaria exposure to develop contextualised health promotions and communication activities such as door-to-door health education and community education campaigns. The health promotions and communication should incorporate a malaria risk map, pictorial information mosquitos and malaria parasite, signs and symptoms of malaria, methods of preventing malaria disease, and where more information can be obtained. This information should also be conveyed via other communication channels such as local television and radio channels, local newspapers, smartphone applications, and in public gathering places such as shopping centres, community centres, cultural centres, religious centres, in public transports and transport stations.

We found out that the lack of electricity was another risk factor that can support malaria transmission. The lack of electricity may result in households spending more time outdoors at night around fires, to cook due to lack of power to operate electric stove, relax and socialise before bedtime. Also, the lack of electricity would result in households sleeping without a fan or an air conditioner which can serve as a form of malaria intervention to ward off mosquitoes. This was revealed in a cohort study conducted in Pakistan aimed at assessing various malaria vector intervention techniques. It was reported that electric fan significantly reduced the total number of Anopheles stephensi and culicine mosquitoes entering the huts, and the amount of blood-fed mosquitoes caught during the study 36. This can be attributed to the fact that fan induces a windy condition which is difficult for mosquitos to fly in, and the windy condition prevents them from landing and blood-feeding on their victim. Also, the wind from fan dilutes and disperses the carbon dioxide humans exhale, which is the main chemical component that attracts mosquitoes to humans. Furthermore, the wind from fan cools off the body of humans, thus reducing body heat, sweat and lactic acid that attracts mosquitoes^37^. With the intermittent load shedding being experienced across SA, households in malaria-endemic areas with electricity would be faced with the risks of malaria associated with lack of electricity. This has the potential to prolong the SA's malaria elimination campaign. Thus, the SA government needs to works closely with ESKOM, SA's main electricity supplier in prioritising the restoration of a steady supply of electricity in malaria-endemic areas. Furthermore, malaria-endemic areas without electricity supply should be prioritised and provided with steady electricity supply and at an affordable rate.

Unemployment was identified as a significant factor of malaria but not as strong as the other socioeconomic variables evaluated in this study. This can be explained by the fact that health care is free in SA 38, thus eliminating the financial burden among those that are unemployed and in pursuance of medical attention. Also, free routine indoor residual spraying is carried out in the endemic areas under active malaria control and surveillance by the KZN malaria program. The spray coverage rate was 82% 8. Nevertheless, its significance can be attributed to the direct relationship between unemployment and the other variables that represent low SES, and in turn, they present a complex interrelationship with malaria disease.

Apart from socioeconomic factors, other demographic factors exhibited significant effects on the risk of malaria in KZN. Regarding gender, both male and female exhibited significant effects on the risk of malaria, but males showed a higher risk of contracting malaria disease. Our study corresponds to previous studies conducted in Tanzania and India that suggests that males are at higher risk of malaria 39, 40. On the contrary, females were discovered to be more susceptible to malaria in Ethiopia 16 and Panama 30. In this present study, gender-specific patterns related to occupations can be attributed to men having a greater risk of contracting malaria disease than women. For example, more men work in fields, forests 30 and travel to neighbouring Kingdom of Eswatini and Mozambique communities of high malaria endemicity for business 41–43. In terms of age group susceptible to malaria in KZN, our findings suggest the elderly are more vulnerable to malaria. This complements previous surveys that suggested older people are more prone to contracting malaria disease 13, 44. Even though the mechanism is not clear, it is assumed that waning immunity with age or low immunity among older people may be responsible. It is worthy of note that the susceptibility and disparity in malaria transmission about gender and age can be attributed to behavioural/ lifestyle and level of immunity, respectively.

Considering the links between low SES and malaria transmission, conceptualising interventions aimed at improving the living conditions on the one hand by SA's department of social development, and on the other hand, the investment of malaria intervention resources (prevention and treatment) by both SA's department of health and social development is vital for sustained poverty alleviation in malaria-endemic communities. This means the provision of malaria intervention resources should be considered as a means of both health intervention and poverty alleviation. This proposed double barrelled-approach and collaborations between both departments can possibly result in sustained malaria elimination. Countries like Greece, Italy, Spain and the USA proved that the double barrelled-approach leads to sustained malaria elimination. They incorporated rigorous anti-malaria intervention, socioeconomics improvement and adequate environmental management strategies. In other words, they modified human dwellings or behaviours to limit human-vector interaction-e.g., improved housing conditions, mosquito proofing of houses, usage of DDT in their indoor residual spraying regime, installation and maintenance of drains and draining of swampy areas to permanently destroy breeding sites 45–47. In the same vein, a randomised controlled trial conducted by Kirby et al. 48 in The Gambia revealed that better housing conditions could significantly reduce the malaria burden. In the randomised controlled trial, house screening (full screening of windows, doors and closing of eaves) revealed that the risk of children contracting malaria disease reduced by 50%.

## Conclusions

The risk of malaria disease exhibits a significant effect in areas or population in a lower socioeconomic bracket. In the local municipalities endemic to malaria in KZN, we discovered that the low socioeconomic status that exhibited the strongest association with malaria risk was lack of toilet facilities after employing the Bayesian multiple regression model. This was followed by no formal education and lack of electricity supply, respectively. However, there are limitations to our study. The spatiotemporal climatic factors that have an influence on malaria transmission were not included in this study, and the Bayesian analysis employed in this study was limited by the short time period, a single year. Thus, the changes in malaria incidence can be masked, and seasonality in malaria incidence could not be ascertained. We suggest robust and long-term studies considering relevant determinants of malaria to provide a clearer picture of malaria transmission, more detailed temporal and spatial information, and the establishment of systems for early warning and early detection of malaria transmission. In addition, a knowledge, attitude, and practices survey are essential. This information will present the relevant policymakers and SA's department of social development and the department of health with reliable information in devising socioeconomic framework that incorporates improved the living conditions, implementing sustainable malaria intervention resources (prevention and treatment), and develop contextualised health promotions, and communication activities. This can ultimately help strengthen the malaria elimination goals in KZN, SA.
